# Multidrug-Resistant Gram-Negative Bacilli: A Retrospective Study of Trends in a Tertiary Healthcare Unit

**DOI:** 10.3390/medicina54060092

**Published:** 2018-11-26

**Authors:** Delia Muntean, Florin-George Horhat, Luminița Bădițoiu, Victor Dumitrașcu, Iulia-Cristina Bagiu, Delia-Ioana Horhat, Dan A. Coșniță, Anca Krasta, Dorina Dugăeşescu, Monica Licker

**Affiliations:** 1 “Victor Babeș” University of Medicine and Pharmacy, Eftimie Murgu Street, No. 2, 300041 Timișoara, Romania; deliacristimuntean@yahoo.com (D.M.); lumiere1tm@yahoo.com (L.B.); vicdumi@yahoo.com (V.D.); bagiuiulia@yahoo.com (I.-C.B.); deliahorhat@yahoo.com (D.-I.H.); dan_andrei.radu@yahoo.com (D.A.C.); dugador06@yahoo.com (D.D.); lickermonica@yahoo.com (M.L.); 2 “Pius Brînzeu” Emergency Clinical County Hospital, Liviu Rebreanu Street, No. 156, 300723 Timișoara, Romania; anca.krasta@yahoo.com; 3 Children Emergency “Louis Turcanu” Timisoara, Iosif Nemoianu Street, No. 2, 300011 Timișoara, Romania

**Keywords:** multidrug-resistant, Gram-negative bacilli, tertiary hospital

## Abstract

*Background and objective:* Bacterial multidrug resistance is particularly common in Gram-negative bacilli (GNB), with important clinical consequences regarding their spread and treatment options. The aim of this study was to investigate the trend of multidrug-resistant GNB (MDR-GNB) in high-risk hospital departments, between 2000–2015, in intervals of five years, with the intention of improving antibiotic therapy policies and optimising preventive and control practices. *Materials and methods:* This is an observational, retrospective study performed in three departments of the most important tertiary healthcare unit in the southwestern part of Romania: the Intensive Care Unit (ICU), the General Surgery Department (GSD), and the Nutrition and Metabolic Diseases Department (NMDD). MDR was defined as acquired resistance to at least one agent in three or more antimicrobial categories. Trends over time were determined by the Cochran–Armitage trend test and linear regression. *Results:* During the study period, a total of 2531 strains of MDR-GNB were isolated in 1999 patients: 9.20% in 2000, 18.61% in 2005, 37.02% in 2010, and 35.17% in 2015. The most significant increasing trend was recorded in the ICU (gradient = 7.63, R² = 0.842, *p* < 0.001). The most common MDR-GNB in the ICU was isolated from bronchoalveolar aspiration samples. Concerning the proportion of different species, most of the changes were recorded in the ICU, where a statistically significant increasing trend was observed for *Proteus mirabilis* (gradient = 2.62, R^2^ = 0.558, *p* < 0.001) and *Acinetobacter baumannii* (gradient = 2.25, R^2^ = 0.491, *p* < 0.001). Analysis of the incidence of the main resistance phenotypes proportion identified a statistically significant increase in carbapenem resistance in the ICU (Gradient = 8.27, R² = 0.866, *p* < 0.001), and an increased proportion of aminoglycoside-resistant strains in all three departments, but more importantly in the ICU and GSD. *Conclusion:* A statistically significant increasing trend was observed in all three departments; the most significant one was recorded in the ICU, where after 2010, carbapenem-resistant strains were isolated.

## 1. Introduction

Bacterial multidrug resistance is particularly common in Gram-negative bacilli (GNB), with important clinical consequences regarding their spread and treatment options.

Over recent years, infections caused by multidrug-resistant (MDR) bacteria have become endemic in many tertiary health care units and hospitals; acquired outbreaks involving such microorganisms are being reported worldwide [1,2,3].

Due to the impact of rising antimicrobial resistance, in 2001, the World Health Organisation (WHO) concluded that high priority should be given to measures that aimed to slow the emergence and reduce the spread of MDR, including ongoing local surveillance programmes and antibiotic stewardship. These measures are particularly important given that the development of antimicrobial agents has been reduced over the last years [4].

Several mechanisms could determine the antimicrobial resistance in GNB. These mechanisms include the enzymatic degradation of antimicrobial agents, such as beta-lactamases in the case of beta-lactam resistance, or modifying enzymes in aminoglycosides resistance. The alteration of antimicrobial targets mediate resistance to fluoroquinolones. Moreover, changes in bacterial membrane permeability can lead to resistance to many antimicrobial agents [5].

Due to the high variability in the prevalence of pathogens and resistance patterns in each hospital, local trends must be identified in order to be able to continually update and increase the effectiveness of empirical and targeted therapies through adapting them to identified microorganisms [6].

The selection of the Intensive Care Unit (ICU), General Surgery Department (GSD), and Nutrition and Metabolic Diseases Department (NMDD) was due to the following criteria: the ICU is the epicentre for the dissemination of MDR strains; surgery combines multiple extrinsic factors which create large breaches in the natural defence systems of the organism, and metabolic pathology is acknowledged to have an intrinsic potential for the increased receptivity to infections. Colonisation strains were not excluded, because colonisation may cause infections, especially in patients with suppressed immune systems or in invasive procedures such as catheters or surgeries [7]. On the other hand, there is also the possibility for the transmission of MDR strains from colonised patients to other people (patients and medical staff) by direct or indirect contact [8].

This study aimed to investigate the trend of MDR-GNB in the most important tertiary health care unit in the southwestern part of Romania over the period 2000–2015, with the intention of improving antibiotic therapy policies and optimising preventive and control practices.

## 2. Materials and Methods

### 2.1. Setting and Study Design

This is an observational, retrospective study performed in three departments of the “Pius Brînzeu” Emergency Clinical County Hospital, Timișoara (PBECCHT): the ICU with 27 beds, the GSD with 70 beds, and the NMDD with 62 beds. To assess the frequency of GNB resistance in the period 2000–2015, we studied four distinct years (in each case for the entire 12-month period of each year: 2000, 2005, 2010, and 2015).

Inclusion into the MDR category was made according to the definition proposed by Magiorakos et al. [9]:

- MDR was defined as acquired resistance to at least one agent in three or more antimicrobial categories;
- XDR was defined as bacterial isolates that remained susceptible to at most two antimicrobial categories.

The authors of this definition identify one of the limitations of these MDR criteria, which also exists for other definitions in the scientific literature, namely that bacterial strains labelled as MDR may possess multiple resistance profile variants. As the differences in MDR interpretation may depend on geographical area and endemicity, based on the *National Programme for the Surveillance and Control of Nosocomial Infections and Monitoring of Antibiotic Use and Antibiotic Resistance* given by the National Institute of Public Health (NIPH) in Romania, we classified the following bacteria that fell within the MDR-GNB category: *Enterobacteriaceae* resistant to extended-spectrum cephalosporins by the production of extended-spectrum beta-lactamases (ESBL) or AmpC, carbapenem-resistant *Enterobacteriaceae*, non-fermenting GNB resistant to ceftazidime or carbapenems [5].

Information was derived from electronic data records provided by the Bacteriology Department of the Central Clinical Laboratory of the hospital and then entered into in an electronic database (Microsoft Excel file) to allow statistical processing. Due to the retrospective nature of the study, based only on the microbiological surveillance data of samples taken during the current medical activity, the informed consent of the patient was not necessary, but the study has received the approval of the PBECCHT Ethics Committee (ref. no. 130/13 Sep. 2017).

### 2.2. Inclusion/Exclusion Criteria

All MDR-GNB from patients admitted to the above-mentioned departments during the study period were included. Patients with an ICU stay under one hour were excluded. Consecutive readmissions were considered in the case of discharged patients who were later readmitted to the same department in the same year. In cases of readmission that were more than one month after the previous discharge, the patient was again included in the total number of patients. A same strain was defined as a strain of the same bacterial species, with the same antibiotic susceptibility pattern, isolated in the same patient during one month, regardless of the samples in which it was isolated, and it was excluded to avoid duplication.

### 2.3. Calculation of Variables

The incidence of MDR-GNB-infected patients was calculated by reporting the number of patients from whom MDR-GNB strains were isolated, and the total number of patients admitted to that department during that year × 100.

The proportion of the biological samples was calculated by reporting the number of specimens from a particular sample from which the MDR-GNB strains were isolated to the total number of samples from which such strains have been isolated × 100.

The proportion of the species was calculated by reporting the number of MDR strains of that species to the total number of MDR-GNB strains isolated × 100.

The percent of phenotypes was calculated by reporting the number of resistant strains with that phenotype, isolated in that department, to the total number of MDR-GNB strains isolated in the same department × 100.

### 2.4. Microbiological Methods

Specimens came from the routinely clinical activity. GNB were isolated on Columbia 5% sheep blood agar (COS-bioMérieux, Marcy l’Etoile, France), lactose-containing Mac Conkey (bioMérieux, France), and chromogenic media for the detection of ESBL production (ESBL Brilliance agar-Oxoid, Basingstoke, UK) and carbapenem resistance (Brilliance CRE agar-Oxoid, UK). For bacterial identification, we used API 20E galleries (bioMérieux, France) or the Vitek automated system (bioMérieux, France). Sensitivity to antimicrobial agents was tested according to the Clinical Laboratory and Standards Institute (CLSI) criteria, by determining the minimum inhibitory concentration (MIC) with the Vitek system, which was supplemented when needed by the disk diffusion method [10].

The phenotypic confirmation of ESBL production was done using the *synergy test* between extended-spectrum cephalosporins and clavulanic acid and *the double-disk synergy test* with cefotaxime, ceftazidime, and cefepime with and without clavulanic acid [10,11]. Carbapenemase production was demonstrated either by the modified Hodge test or by combined disc methods (KPC, MBL, and OXA-48 Confirm kit, Rosco Diagnostica, Denmark) [10,12,13].

### 2.5. Statistical Analysis

The categorical variables, which were characterised by number and percentage, were compared by applying Pearson’s Chi-square with Fisher’s exact test. Trends over time were determined by the Cochran–Armitage trend test and linear regression. Statistical significance was calculated by two-tailed tests, and the significance threshold was set at *p* values ≤ 0.05. The statistical analysis of the database was performed using the SPSS version 20 (Armonk, NY, USA: IBM Corp) and XLSTAT (New York, NY, USA: Addinsoft SARL).

## 3. Results

After monitoring a total of 7040 patients admitted in the three above-mentioned departments in 2000, 6245 in 2005, 5891 in 2010, and 6068 in 2015, 2531 strains of MDR-GNB were isolated from 1999 patients (184 in 2000, 372 in 2005, 740 in 2010, and 703 in 2015), with an incidence trend shown in Table 1.

An increasing trend was observed that was statistically significant in all three departments, but with obvious differences. The most significant increase was recorded in the ICU (gradient = 7.63, R² = 0.842) followed by a significant linear increase in the NMDD (gradient = 2.58, R² = 0.952). In the GSD, the rising trend was much lower.

MDR-GNB were most commonly isolated in the ICU from bronchoalveolar aspiration samples. After a statistically significant decrease in 2005 versus 2000 (*p* < 0.001), the percentage of bronchoalveolar aspiration (among the samples from which MDR-GNB were isolated) significantly increased in 2010 versus 2005 (*p* < 0.001), remaining at the same percentage in 2015 (*p* = 0.669). The decrease in 2005 was due to an increased proportion in the blood cultures, with a peak in that year. In 2010, we recorded a significant decrease (*p* < 0.001), followed by an increase in percentage in 2015 in comparison to 2010 (*p* < 0.001). In the same department also in 2005, catheter-associated infections reached a peak of 7.14% (Table 2).

**Figure 1:**
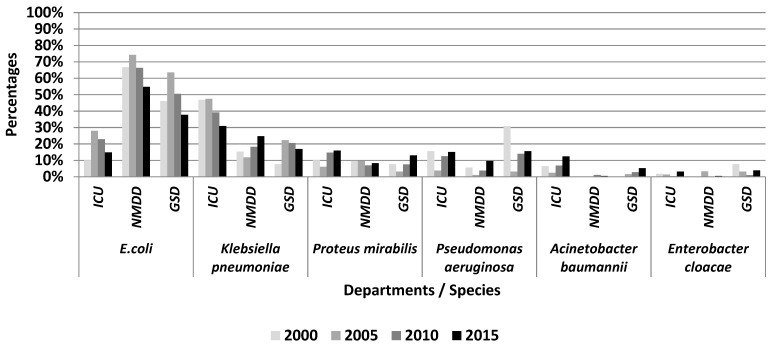
The proportion of species among MDR-GNB isolated included in this study.

Concerning the proportion of different species in MDR *Enterobacteriaceae* and non-fermenting bacteria (Figure 1), most of the changes were recorded in the ICU. Thus, a statistically significant increasing trend was observed for *Proteus mirabilis* (gradient = 2.62, R^2^ = 0.558, *p* < 0.001) and *Acinetobacter baumannii* (gradient = 2.25, R^2^ = 0.491, *p* < 0.001), while the proportion of *Klebsiella pneumoniae* registered a very significant decreasing trend (gradient = −5.60, R² = 0.866, *p* < 0.001).

In the GSD, *E. coli* had a statistically significant decreasing trend (gradient = −3.85, R^2^ = 0.213, *p* = 0.014). Although for *Proteus mirabilis* the increasing statistical trend was only at the statistical significance threshold (gradient = 2.02, R² = 0.420, *p* = 0.065), this increase is important from an epidemiological and a clinical point of view, due to the links with the ICU.

On the NMDD, *E. coli* recorded a significantly decreasing trend (gradient = −4.35, R² = 0.492, *p* = 0.004), while *Klebsiella pneumoniae* had an increased trend (gradient = 3.45, R² = 0.671, *p* = 0.011).

The strains of *Pseudomonas aeruginosa* and *Enterobacter cloacae* did not portray statistically significant trends in any department; the high percentage of *Pseudomonas aeruginosa* strains was due to the reduced MDR-GNB number identified in the GSD in 2000.

The analysis of the incidence of the main beta-lactam resistance phenotypes only identified a statistically significant increase in carbapenem resistance in the ICU (gradient = 8.27, R² = 0.866, *p* < 0.001), which is presented in Table 3.

Concerning the other resistance phenotypes, an increased proportion of aminoglycoside-resistant strains in all three departments was noticed, but more importantly in the ICU and GSD, as highlighted in Table 4. The proportion of fluoroquinolone-resistant strains varied with statistical significance in the ICU, but the trend was not linear, while the percentage of resistance to folate pathway inhibitors and tetracyclines did not vary during the study in any of the three departments. None of the strains included in the study showed acquired resistance to glycylcyclines or polymyxins.

## 4. Discussion

This study had its starting point from the emergence of GNB in nosocomial infections with steadily rising trends over the past decade in Europe. Another important reason for conducting this study was the evidence of a risk of increased mortality among patients with hospital-acquired infections (HAI), in the event of a failure to control the pathogen during the first 24–48 h [14,15,16].

Under conditions of the increased use of invasive devices and procedures, aggressive antibiotic therapies and the increased number of more elderly patients undergoing surgery, treated with immunosuppressive therapies, or with co-morbidities causing immune deficiencies, we initiated this study in a tertiary healthcare unit with over 1000 beds, 11 medical and 12 surgical departments, and the most important ICU in southwest Romania.

Data from other studies that were conducted during the same period suggested a possible return in Europe to the situation where GNB dominated the etiology of systemic infections, which was a position that they lost in 1980 in favor of Gram-positive (GP) pathogens, especially *Staphylococcus* [17,18,19,20]. This change raises concerns, taking into account the higher mortality of sepsis caused by GNB as compared with GP cocci and the continual emergence of resistance among these bacterial species [3].

Based on the data obtained, we observed a decreased isolation of *Klebsiella pneumoniae*, which was concomitant with the emergence of *Proteus mirabilis* and *Acinetobacter baumannii* strains in the ICU. *Acinetobacter baumannii* is frequently involved in the etiology of assisted post-ventilation pneumonia, post-operative wound infections, septicemia and meningitis associated with ventricular artery disease, and ulcers pressure infections. Furthermore, the increased incidence of MDR strains requires the use of carbapenems, as well as in the case of resistance to this class, the use of colistin and tigecycline [21,22,23,24,25]. Parallel growth in the incidence of colistin-resistant *Enterobacteriaceae*, as *Proteeae*, increase the risk of inefficiency of these backup antimicrobial agents in case of extensively drug resistant (XDR)-*Acinetobacter baumannii* and *Proteus* spp. The impact of this upward trend is quantified in excess of morbidity, fatality, prolongation of hospitalisation, and significant additional financial costs for the ICU [26]. Additionally, there is an increase in the epidemiological risk of spreading these strains in other departments, affecting the quality of medical services provided by the hospital.

Most of the MDR-GNB strains were isolated from bronchial aspirates in ICU patients, from wound secretions of GSD patients, and from the urine of NMDD patients; these results are comparable with other studies conducted during the same period [27,28,29]. In our study, in 2005, in accordance with other reports, MDR-GNB were frequently isolated from blood cultures in the ICU and GSD [30,31,32,33].

The European Centre for Disease Prevention and Control (ECDC) data from 2007 showed high levels of resistance to third generation cephalosporins, fluoroquinolones, and aminoglycosides in southeastern European countries, especially for *Klebsiella pneumoniae* and *Pseudomonas aeruginosa* isolates [34].

In 2009, ECDC data highlighted the increase in MDR-GNB strains, which limited the number of therapeutic options, especially as a few countries in this period have reported a high proportion of carbapenem resistance in the case of *Klebsiella pneumoniae* [35].

In regard to our country, carbapenem resistance and MDR-GNB invasive isolates places Romania in the first place among European Antimicrobial Resistance Surveillance Network (EARS Net) countries [36]. In another study that was conducted in the same unit between 2012–2013, the incidence density of resistant strains was 7.88 for ESBL producing *Klebsiella pneumoniae*, 4.17 for *Proteus mirabilis*, and 4.68/1000 patient-days for MDR-*Acinetobacter baumannii* [26]. 

The increased resistance among GNB is frequently related to the high selective pressure of antimicrobials that are commonly used in hospitals [37,38,39]. The inclusion of gentamycin in many protocols of postoperative antibioprophylaxis (especially in digestive, urological, and gynecological surgery), as well as its frequent use in antibiotherapy, has led to increased selective pressure and incidence of aminoglycoside-resistant strains [40]. Correspondingly, in this study, all three departments that we observed showed an increase in the proportion of aminoglycoside-resistant strains.

However, frequent ESBL or cephalosporinases-producing strains did not show statistically significant trends in any department studied.

Resistance to fluoroquinolones did not increase significantly, but in the ICU, it changed significantly within the four years.

Also in the ICU, XDR strains have been isolated, which are characteristic for this department, where the accumulation of intrinsic and extrinsic nosocomial factors favors the selection of such bacterial strains.

The upward trend of the MDR and XDR strains has multiple causes: prolonged postoperative antibiotic prophylaxis in Romanian hospitals, a high antibiotic consumption rate (especially those with broad spectrum) both in hospitals and the community, a lack of updating clinical protocols depending on local circulatory trends, reduced the possibilities of isolation in high-risk departments, as well as reduced multidisciplinarity in the case of management and limited collaboration between clinicians, microbiologists, and epidemiologists [41]. This affects the ability of hospitals to offer optimal medical treatment, especially in high-risk departments, such as the ICU, surgery, and departments designed to treat immunosuppressed patients.

The increasing trend of resistance was seen in the majority of the bacteria studied in other European ICUs [42].

Worldwide, we are witnessing the emergence of MDR strains that may severely compromise the survival chances of patients. The key interventions against this phenomenon are appropriate infection control measures and the more considered use of antimicrobial agents [2]. To save the existing potential antibiotics, the government and physicians should limit the antibiotic prescription to prevent antibiotic resistance; for that reason, people should not easily get access to self-medicated antibiotics. Although antibiotics are released by prescription, self-medication is still present in the community environment, which raises the consumption of antibiotics. In Romania, there is the need for stricter monitoring of bacterial multidrug resistance, both locally and nationally, and studies such as this one give a complete overall picture and contribute to prioritising the topic in public health policies.

In the tertiary healthcare unit studied in this paper, following the increased levels of bacterial resistance, particularly in the ICU, a restrictive antibiotic therapy policy was adopted in 2005, and in 2011 the *Committee for the prevention of healthcare-associated infections* was created in the PBECCHT. Efforts initially focussed on the identification and annihilation of exogenous MDR sources by performing microbiological cultures of samples collected from all potentially contaminated medical devices or from other objects in the hospital environment and on the revision of all nosocomial risk procedures. In parallel, measures to prevent cross-contamination were taken, which can also be found in the guidelines for the prevention and control of MDR-GNB [43,44]. 

The design limitations of the present study include: retrospective character, discontinuous analysis of the three departments, at five-year intervals, and the involvement of a single tertiary medical unit and a single university center. Also, the lengthy investigation period generated inherent variations in MDR-GNB strains identification methodology, terminology, prevention, and control interventions applied in the hospital. As well as that, the interpretation of resistance phenotypes was done based on results published by the Bacteriology Department, without the opportunity to add genotyping methods for MDR strains.

Despite these, the study adds information to the data reported worldwide regarding the incidence of MDR-GNB in patients of high-risk hospital departments. Furthermore, the complete implementation of prevention and control methods is essential for the limitation of MDR-GNB dissemination and prevention of their further spread in the community.

## 5. Conclusions

A statistically significant increasing trend of MDR-GNB was observed in all three departments, with the most significant one recorded in the ICU, where most of the recorded changes targeted *Proteus mirabilis* and *Acinetobacter baumannii* strains. The same increasing trend was recorded for *Proteus mirabilis* in GSD and *Klebsiella pneumoniae* in NMDD. Analysis of the incidence of the main resistance phenotypes identified a statistically significant increase in carbapenem resistance in the ICU and an increased proportion of aminoglycoside-resistant strains in all three departments.

## Figures and Tables

**Table 1:** Incidence of infected and colonised patients with multidrug-resistant Gram-negative bacilli (MDR-GNB). ICU: Intensive Care Unit, GSD: General Surgery Department, NMDD: Nutrition and Metabolic Diseases Department.

Departments	2000 *n* (%) *	2005 *n* (%) *	2010 *n* (%) *	2015 *n* (%) *	Gradient	R²	*p* *
ICU	100 (3.73)	221 (10.74)	455 (26.18)	422 (24.03)	7.63	0.842	<0.001
GSD	13 (0.56)	61 (2.80)	104 (5.06)	73 (3.08)	0.98	0.474	<0.001
NMDD	71 (3.47)	90 (4.47)	181 (8.63)	208 (10.67)	2.58	0.952	<0.001
Total	184 (2.61)	372 (5.96)	740 (12.56)	703 (11.58)	3.35	0.842	<0.001

* Cochran–Armitage trend test.

**Table 2:** Proportion of various biological samples from which MDR-GNB have been isolated.

Biological Specimens	2000			2005			2010			2015		
	ICU *n* (%) *	GSD *n* (%) *	NMDD *n* (%) *	ICU *n* (%) *	GSD *n* (%) *	NMDD *n* (%) *	ICU *n* (%) *	GSD *n* (%) *	NMDD *n* (%) *	ICU *n* (%) *	GSD *n* (%) *	NMDD *n* (%) *
Bronchoalveolar lavage	80 (72.07)	0 (0)	1 (1.38)	128 (39.75)	1 (1.58)	0 (0)	434 (58.88)	5 (4.63)	6 (3.17)	371 (60.03)	0 (0)	0 (0)
Sputum	1 (0.9)	0 (0)	1 (1.38)	1 (0.31)	1 (1.58)	3 (3.19)	17 (2.3)	1 (0.92)	7 (3.7)	15 (2.42)	1 (1.26)	20 (9.09)
Endotracheal aspirate	2 (1.8)	1 (7.69)	1 (1.38)	8 (2.48)	3 (4.76)	3 (3.19)	0 (0)	0 (0)	0 (0)	2 (0.32)	0 (0)	0 (0)
Blood	0 (0)	0 (0)	0 (0)	44 (13.66)	4 (6.35)	0 (0)	22 (2.98)	0 (0)	0 (0)	45 (7.28)	0 (0)	0 (0)
Catheter tip	0 (0)	0 (0)	0 (0)	23 (7.14)	1 (1.58)	0 (0)	34 (4.61)	1 (0.92)	0 (0)	19 (3.07)	1 (1.26)	0 (0)
Wound secretion	17 (15.31)	7 (53.84)	4 (5.55)	26 (8.07)	32 (50.79)	12 (12.76)	67 (9.09)	63 (58.33)	32 (16.93)	33 (5.33)	31 (39.24)	28 (12.72)
Urine	11 (9.9)	4 (30.77)	64 (88.89)	72 (22.36)	17 (26.98)	76 (80.85)	138 (18.72)	16 (14.81)	142 (75.13)	114 (18.44)	19 (24.05)	165 (75)
Other *	0 (0)	1 (7.69)	1 (1.38)	20 (6.21)	4 (6.35)	0 (0)	25 (3.39)	22 (20.37)	2 (1.05)	19 (3.07)	27 (34.18)	7 (3.18)
Total	111 (100)	13 (100)	72 (100)	322 (100)	63 (100)	94 (100)	737 (100)	108 (100)	189 (100)	618 (100)	79 (100)	220 (100)

* Other = cerebrospinal fluid; puncture fluids; abscess pus; pressure ulcer secretion; drainage tube.

**Table 3:** The proportion incidence of beta-lactam resistance phenotypes among MDR-GNB.

Department	Phenotypes	2000 *n* (%)	2005 *n* (%)	2010 *n* (%)	2015 *n* (%)	Gradient	R²	*p* *
ICU	CASE	30 (27.52)	177 (59.59)	509 (73.55)	295 (49.00)	7.84	0.272	0.727
	ESBL	79 (72.47)	120 (40.40)	183 (26.44)	307 (50.99)	−7.84	0.272	0.727
	CR	0 (0)	0 (0)	69 (9.97)	146 (24.25)	**8.27**	0.866	**<0.001**
Total		109 (100)	297 (100)	692 (100)	602 (100)			
GSD	CASE	10 (76.92)	51 (80.95)	100 (93.45)	64 (83.11)	2.51	0.251	0.474
	ESBL	3 (23.07)	12 (19.04)	7 (6.54)	13 (16.88)	−3.11	0.324	0.474
	CR	0 (0)	0 (0)	1 (0.93)	1 (1.29)	0.48	0.888	0.366
Total		13 (100)	63 (100)	107 (100)	77 (100)			
NMDD	CASE	59 (81.94)	84 (90.32)	174 (93.04)	193 (88.12)	2.13	0.337	0.349
	ESBL	13 (18.00)	9 (9.67)	13 (6.95)	26 (11.87)	−5.51	0.966	0.349
	CR	0 (0)	0 (0)	1 (0.53)	1 (0.45)	0.19	0.726	0.462
Total		72 (100)	93 (100)	187 (100)	219 (100)			
**Total**		**194**	**453**	**986**	**898**			

* Cochran–Armitage trend test; CASE: cephalosporinase hyperproduction, ESBL: extended spectrum beta-lactamases, CR: carbapenem resistance.

**Table 4:** The evolution of aminoglycosides, fluoroquinolones, folate pathway inhibitors, glycylcyclines, polymyxins, and tetracyclines resistance proportion among MDR-GNB.

Department	Antibiotic	2000 *n* (%)	2005 *n* (%)	2010 *n*(%)	2015 *n* (%)	Gradient	R²	*p* *
**ICU**	Aminoglycosides	47 (43.11)	173 (58.24)	576 (83.23)	517 (85.88)	**15.33**	0.928	<0.001
	Fluoroquinolones	73 (66.97)	119 (40.06)	283 (40.89)	404 (67.10)	0.122	0.0001	<0.001
	Folate pathway inhibitors	67 (61.46)	181 (60.94)	417(60.26)	382(63.45)	0.529	0.558	0.449
	Glycylcycline	0 (0)	0 (0)	0 (0)	0 (0)	NA	NA	NA
	Polymyxins	0 (0)	0 (0)	0 (0)	0 (0)	NA	NA	NA
	Tetracyclines	59 (54.12)	167 (56.22)	392 (56.64)	326 (54.15)	0.051	0.002	0.687
Total		109 (100)	297 (100)	692 (100)	602 (100)			
**GSD**	Aminoglycosides	3 (23.07)	44 (69.84)	98 (91.58)	64 (83.11)	**20.186**	**0.727**	<0.001
	Fluoroquinolones	3 (23.07)	16 (25.39)	25 (23.36)	24 (31.16)	2.224	0.585	0.398
	Folate pathway inhibitors	6 (46.15)	31 (49.20)	47 (43.92)	34 (44.15)	−1.128	0.355	0.606
	Glycylcycline	0 (0)	0 (0)	0 (0)	0 (0)	NA	NA	NA
	Polymyxins	0 (0)	0 (0)	0 (0)	0 (0)	NA	NA	NA
	Tetracyclines	4 (30.76)	17 (26.98)	29 (27.10)	32 (41.55)	3.249	0.373	0.093
Total		13 (100)	63 (100)	107 (100)	77 (100)			
**NMDD**	Aminoglycosides	55 (76.38)	60 (64.51)	167 (89.3)	190 (86.75)	**5.59**	**0.408**	<0.001
	Fluoroquinolones	19 (26.38)	33 (35.48)	53 (28.34)	61 (27.85)	−0.273	0.007	0.913
	Folate pathway inhibitors	44 (61.11)	59 (63.44)	108 (57.75)	129 (58.90)	−1.232	0.399	0.545
	Glycylcycline	0 (0)	0 (0)	0 (0)	0 (0)	NA	NA	NA
	Polymyxins	0 (0)	0 (0)	0 (0)	0 (0)	NA	NA	NA
	Tetracyclines	50 (69.44)	62 (66.66)	117 (62.56)	144 (65.75)	−1.517	0.477	0.592
Total		72 (100)	93 (100)	187 (100)	219 (100)			
**Total**		**194**	**453**	**986**	**898**			

* Cochran–Armitage trend test; NA = Not applicable.
